# Filoviruses are ancient and integrated into mammalian genomes

**DOI:** 10.1186/1471-2148-10-193

**Published:** 2010-06-22

**Authors:** Derek J Taylor, Robert W Leach, Jeremy Bruenn

**Affiliations:** 1 Department of Biological Sciences, The State University of New York at Buffalo, Buffalo, NY 14260, USA; 2 Center for Computational Research, The State University of New York at Buffalo, Buffalo, NY 14203, USA

## Abstract

**Background:**

Hemorrhagic diseases from Ebolavirus and Marburgvirus (Filoviridae) infections can be dangerous to humans because of high fatality rates and a lack of effective treatments or vaccine. Although there is evidence that wild mammals are infected by filoviruses, the biology of host-filovirus systems is notoriously poorly understood. Specifically, identifying potential reservoir species with the expected long-term coevolutionary history of filovirus infections has been intractable. Integrated elements of filoviruses could indicate a coevolutionary history with a mammalian reservoir, but integration of nonretroviral RNA viruses is thought to be nonexistent or rare for mammalian viruses (such as filoviruses) that lack reverse transcriptase and replication inside the nucleus. Here, we provide direct evidence of integrated filovirus-like elements in mammalian genomes by sequencing across host-virus gene boundaries and carrying out phylogenetic analyses. Further we test for an association between candidate reservoir status and the integration of filoviral elements and assess the previous age estimate for filoviruses of less than 10,000 years.

**Results:**

Phylogenetic and sequencing evidence from gene boundaries was consistent with integration of filoviruses in mammalian genomes. We detected integrated filovirus-like elements in the genomes of bats, rodents, shrews, tenrecs and marsupials. Moreover, some filovirus-like elements were transcribed and the detected mammalian elements were homologous to a fragment of the filovirus genome whose expression is known to interfere with the assembly of Ebolavirus. The phylogenetic evidence strongly indicated that the direction of transfer was from virus to mammal. Eutherians other than bats, rodents, and insectivores (i.e., the candidate reservoir taxa for filoviruses) were significantly underrepresented in the taxa with detected integrated filovirus-like elements. The existence of orthologous filovirus-like elements shared among mammalian genera whose divergence dates have been estimated suggests that filoviruses are at least tens of millions of years old.

**Conclusions:**

Our findings indicate that filovirus infections have been recorded as paleoviral elements in the genomes of small mammals despite extranuclear replication and a requirement for cooption of reverse transcriptase. Our results show that the mammal-filovirus association is ancient and has resulted in candidates for functional gene products (RNA or protein).

## Background

The ongoing threat of emerging hemorrhagic diseases has made the search for reservoir species with a history of coevolution with filoviruses a priority [1,2]. Outbreaks of filovirus infections are known from Africa and the Phillipines [3-5] and, in some cases, the mortality of primates is so severe as to raise concerns of extinction [5]. Bats are considered a candidate for a reservoir based on the detection of filovirus-specific RNA, antibodies, and viral particles [1,6-10]. Still, the average seroprevalence in tested bats is much smaller than expected (usually < 5%) for large colonies of a main reservoir [6], and the ability of bats to maintain a persistent hypovirulent infection is unknown. Rodents and insectivores (shrews) have further been proposed as the leading candidates for filovirus reservoirs by modeling, the detection of filovirus RNA, and in one specimen, the potential detection of a DNA copy [2,11]. Rodents (mice and guinea pigs) share one expected feature of coevolution -- asymptomatic infections from wild-type filoviruses [12]. However, a reservoir role for rodents and shrews has been questioned because only one study has detected filovirus RNA fragments in these small mammals, and many more outbreaks than observed are expected from a rodent reservoir that is commensal with humans [7]. Moreover, no live viruses, filovirus particles or antibodies to filoviruses have been found in rodents or shrews. Distinguishing principal reservoir species from "spillover" infections remains a challenge.

**Figure 1:**
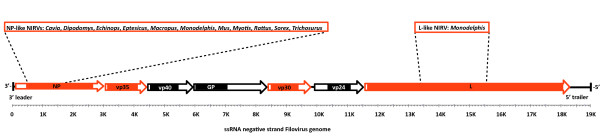
**Genome map of a filovirus showing the gene order and regions of homology with proposed filovirus-like elements in mammals**. Dashed lines indicate the boundaries of the non-retroviral integrated RNA virus elements (NIRVs) and depict a bias for the N-terminal region of the NP gene. Mammalian genera that show homology with a gene of filovirus are listed above the genome map. Solid colors within the coding region arrows indicate the size of the product. Red shading indicates proteins associated with the viral RNA in the ribonucleoprotein complex.

Filoviruses are a family of non-segmented negative sense RNA viruses with filamentous virions (Fig. 1). The protein-coding genes in the filovirus genomes (3'-NP, VP35, VP40, GP, VP30, VP24, and L protein-5') have a transcriptional gradient from NP to L protein [13]. The two major evolutionary groups of filoviruses have been assigned to the genera *Ebolavirus *and *Marburgvirus*. Filoviruses are estimated to have diverged for less than 10,000 YA [14]--about the same timescale as the rise of agriculture. Although high mutation rates in RNA viruses have shrouded nearly every interfamilial relationship, the Order Mononegavirales, which contains Filoviridae, is an exception [13]. Here, filoviruses show significant sequence similarity to some of the Paramyxoviridae such as *Morbillivirus *(e.g. measles and Rinderpest viruses) [15]. Notably, the N-terminal 450 amino acid residues of NP, which is examined in the present study, shows significant conservation among the Mononegavirales and is needed for self-assembly of the nucleoprotein [16].

There are now several cases in eukaryotes where non-retroviral integrated RNA viruses (NIRVs) have been detected [17,18]. Still, this type of transfer is believed to be extremely rare in mammals [17,19] because the process requires the cooption of reverse transcriptase and perhaps replication within the nucleus. The sole mammalian example is bornavirus, which is unique among RNA viruses of animals in developing persistent infections within the nucleus. The study of NIRVs requires an evolutionary approach where the direction of transfer is tested. Evolutionary comparisons among NIRVs have been carried out for the Totiviridae in yeast [20], and the Bornaviridae in mammals [17]. In the Totivirus system there strong support for the direction of transfer from virus to fungus, and a role for the expression of NIRVs in viral interference has been proposed [20]. We proposed that NIRVs are more common than presently known and might be detected in other systems with persistent infections of non-retroviral RNA viruses. As part of a search for NIRVs in NCBI databases we found strong BLAST matches of NP sequences from filoviruses to translated genomic sequences from small mammals. We aimed to test if these sequence similarities might indicate NIRVs of filoviruses.

## Results and Discussion

**Figure 2:**
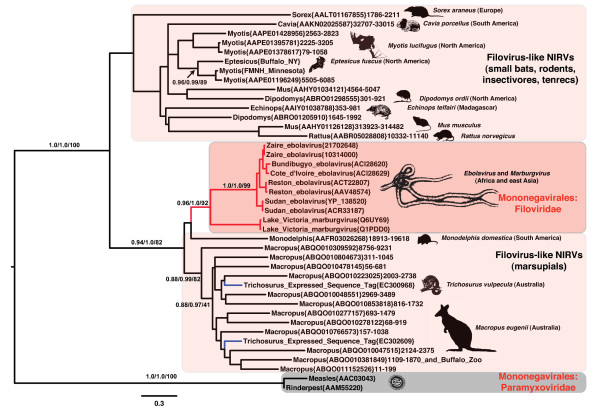
**Midpoint rooted maximum likelihood phylogram of nucleoprotein (NP) amino acid sequences from filoviruses, morbilliviruses and related mammalian genomic and EST sequences**. Branches with more than two sequences and strong support (at least 90 for bootstrap or 95 for Bayesian posterior probability) have values shown above the branch (in the order of approximate likelihood ratio tests, Bayesian Posterior Probabilities, and non-parametric bootstrap values). Parentheses contain GenBank Accession numbers and are followed by the range of the sequence for nucleotide submissions. Red filled branches indicate clades of viruses (Mononegavirales), black filled branches indicate mammalian sequences, and blue filled lines indicate expressed sequence tags. Geographic origins are given in parentheses adjacent to species names. Shaded cartoons indicate outlines of species represented in the analysis.

tBLASTn with Marburgvirus NP amino acid sequence yielded matches with low expect values (as low as 10^-49^), indicating that similarity is unlikely to be a chance result. We found twenty matches with expect values less than the standard "significance" value of 10^-5 ^(see Fig. 2). The tammar wallaby, (*Macropus eugenii*) showed the strongest similarity (49.4% identity) and also had at least 12 different strong sequence matches. The little brown bat (*Myotis lucifugus*) had four significant matches, while the guinea pig (*Cavia porcellus*), Ord's kangaroo rat (*Dipodmys ordii*), the common shrew (*Sorex araneus*), and the gray short-tailed opossum (*Monodelphis domestica; *Chromosome 2) each had single matching sequences with expect values <10^-5^. Another marsupial, the common brushtail possum (*Trichosurus vulpecula*) had six strong matches from the Expressed Sequence Tags (EST) database. All but three of these sequences (including the EST matches) had at least one apparent disruption of the open reading frame (ORF). tBLASTn with the L protein yielded one value with a low expect value (10^-74^), the gray short-tailed opossum (*Monodelphis domestica; *Chromosome 3). A tBLASTn search using the best matching placental mammal match from the original NP search as a query sequence also yielded strong matches in mammals: the pygmy hedgehog tenrec (*Echinops telfairi*), the mouse (*Mus musculus*) and the brown rat (*Rattus norvegicus*). The filovirus-like EST nucleotide sequences from the common brushtail possum had a BLAST match to a single region of the wallaby genome with longest match (DY609334) having a 78% identity (9% of mismatches are gaps) for 662 bases.

We tested for integrated DNA based copies of the filovirus-like sequences in the two mammals with the most copies, the tammar wallaby and the little brown bat. We designed PCR primers from mammalian genomic sequence flanking the longer BLAST matches and carried out PCR amplification of DNA extractions from different specimens than used for existing genome projects. Our sequence of the tammar wallaby had only a single transition difference from the genome project sequence. The sequence of the little brown bat from Minnesota (FMNH 172384) had a similarity of 96% with four indels compared to contig (AAPE01196249) from the existing genome. To test for the presence of a filovirus-like DNA sequence in an additional insectivorous bat, we extracted DNA from a specimen of big brown bat (*Eptesicus fuscus*). Using primers designed from the little brown bat, we again obtained PCR product and sequence. In this case, the identity between the sequences of the two genera of bats was 87% with 11 indels. In each case the similarity of the new sequences obtained from DNA to genomic sequence is consistent with an integrated filovirus-like DNA copy in these mammalian genomes.

**Figure 3:**
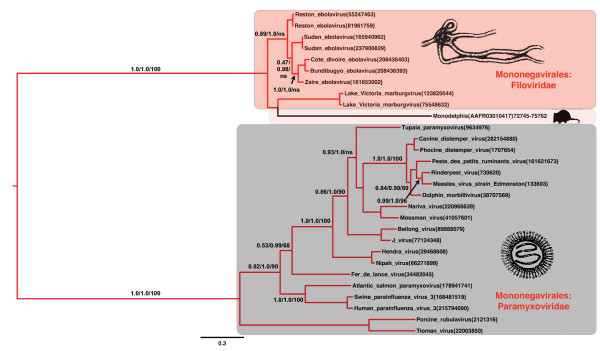
**Midpoint rooted maximum likelihood phylogram of L protein amino acid sequences from filoviruses, Paramyxoviridae, and a South American marsupial genomic sequence**. Labeling and shading details are as in Fig. 2 except that the species name and continent for the mammalian sequence are provided in the caption: *Monodelphis domestica *(South America).

**Figure 4:**
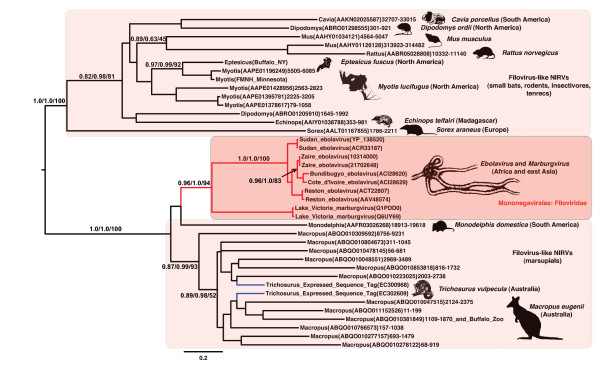
**Midpoint rooted maximum likelihood phylogram of nucleoprotein (NP) amino acid sequences from filoviruses and related mammalian genomic and EST sequences showing the paraphyly of mammals**. Branches with more than two sequences and strong support (at least 90 for bootstrap or 95 for Bayesian posterior probability) have values shown above the branch (in the order of approximate likelihood ratio tests, Bayesian Posterior Probabilities, and non-parametric bootstrap values). Parentheses contain GenBank Accession numbers and are followed by the range of the sequence for nucleotide submissions. Red filled branches indicate clades of viruses (Mononegavirales), black filled branches indicate mammalian sequences, and blue filled lines indicate expressed sequence tags. Geographic origins are given in parentheses adjacent to species names. Shaded cartoons indicate outlines of species represented in the analysis.

We next carried out a phylogenetic analysis of the NP and L protein amino acid sequence alignments with Mononegavirales (paramyxovirids and filovirids) to assess the direction of the transfer. Because the L protein gene is known to be the most conserved gene in the Mononegavirales, a large number of BLAST matches with expect values <10^-5 ^was found between the families of Mononegavirales in L protein compared to the NP. The midpoint rooted maximum likelihood (ML) phylogram placed the potential mammalian NIRVs within the Mononegavirales, and revealed that the mammalian sequences are more closely related to filoviruses than to Paramyxoviruses (Figs. 2, 3). Indeed the L protein-like sequence from *Monodelphis *was more closely related on the best ML tree to *Marburgvirus *than to other known filoviruses (i.e., *Ebolavirus*) (Fig. 3). This result suggests that the most recent integration of filoviruses from our data involves South American marsupials. The NP analysis also revealed that the South American *Monodelphis *is more closely related to known filoviruses than to other mammalian sequences (Fig. 3 and Additional file 1: Fig. S1). Although many of the sequences are of different lengths in the NP alignment (Additional file 2: Fig. S2), it is now well known that sequences of very different lengths can be accurately placed on phylogenies [21]. However, there could be long-branch effects or alignment effects for the NP phylogeny as the exclusion of the distantly related *Morbillivirus *sequences yielded the same mammalian paraphyly, but increased the support values (Fig. 4). For both genes, the placement of the mammalian NIRVs with the filoviruses (i.e. within Mononegavirales) had maximum support for each measure of reliability. The placement and the strong support values for this node are consistent with the direction of transfer from viruses (Mononegavirales) to mammalian genomes. Endogenous reverse transcriptase activity has been shown experimentally to integrate non-retroviral RNA viruses in mammals [17,22] and may have played a role in filovirus integration. Interestingly, the closest flanking coding regions of integrated filovirus-like elements to at least five of the NIRV's of *Macropus*, and the separate NP and L-like NIRVs of *Monodelphis*, are truncated or disrupted non-LTR retrotransposons of the LINE-1 family. Our results represent the first case of NIRV formation in mammals with a virus that has extranuclear replication [17].

The observation that most of the mammalian sequences have ORF disruptions and possess only truncated NP-like genes (Fig. 1) is also inconsistent with a transfer from mammals to virus. Only *Monodelphis *has more than one different filovirus-like gene (Additional file 3: Fig. S3) and these (the NP and L protein-like sequences) are on separate chromosomes. The apparent genic bias of NIRVs for the NP gene could have a biological explanation. Because of the transcription gradient in the Mononegavirales, the most common primary transcript is NP [13]. We also note that experimental expression of an N-terminal portion of the *Ebolavirus *NP gene (from residue 1-450 in wildtype NP) that is positionally homologous to the region of NP spanned by mammalian NIRVs (from residue 18-405 in wildtype NP, NP_066243) is sufficient to inhibit the formation of *Ebolavirus *minigenomes in a dosage specific fashion [23]. A background transcription bias could account for overrepresentation in NIRVs of NP, but such a bias fails to explain the N-terminal bias within the NIRVs of NP. The bias is consistent with the experimental filoviral interference mechanism involving the N-terminal of NP.

**Figure 5:**
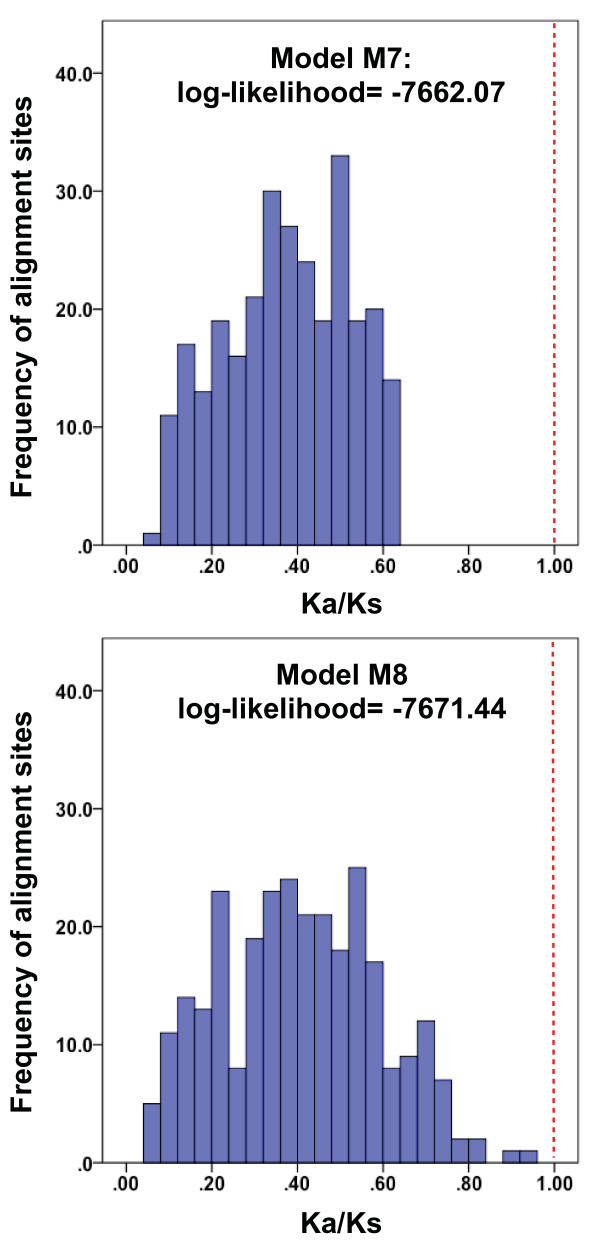
**Histograms of K_a_/K_s _values calculated from alignment sites of the filovirus-like elements in eleven species of mammals**. Values are calculated using Bayesian methods and a model that accommodates neutral, positive and negative selection (M8 below), and a model that accommodates largely negative or purifying selection (M7 above). Note the better fit of the purifying selection model. Red dashed lines indicate the expected values under neutral evolution for non-functional pseudogenes, while values <<1 are consistent with purifying selection.

Despite ORF disruptions, it is clear that at least some mammalian filovirus-like NIRVs of NP are expressed. In the marsupial *Trichosurus*, we detected six different NP-like ESTs (EC302609, DY609334, EC300968, EC310159, DY613238, EC352436) from three tissue-specific cDNA libraries: liver, spleen/lymphatic system and gonads. These tissues play an important role in the pathology and replication of filoviruses [24]. We did not detect the NIRV in the cDNA libraries made from brain, whole embryo, kidney, uterus/reproductive tract, or gut tissues. Still, non-functional pseudogenes can be transcribed by interactions with neighboring functioning loci [25]. We tested for selective maintenance of codon structure in the filovirus-like NIRVs as a further indication of function. Comparisons of rates of amino-acid changing substitutions (d_N _or K_a_) to rates of silent substitutions (d_S _or K_s_) do bear the signature of selective codon maintenance or purifying selection. Non-functional regions should conform to neutral expectations where d_N _= d_S _and d_N_/d_S _= 1 [26]. For regions undergoing purifying selection, the silent substitution rate should prevail whereby d_N_-d_S _<< 0 and d_N_/d_S _<< 1. The codon-based test of neutrality using the model of Kumar (which accommodates transition/transversion rate bias) indicates that silent mutations are significantly overrepresented in an alignment of filovirus-like NIRVs (d_N_-d_S _= -9.427, P < 0.001) [27]. Likewise, Bayesian calculations of site-specific K_a_/K_s _using evolutionary models that accommodate codon usage differences [28], reveal a prevailing pattern of values significantly less than 1 (Fig. 5). Under a model that allows purifying, neutral and positive selection (Model M8), the distribution of K_a_/K_s _peaks at about 0.4. For the M8 model, 67 percent of these alignment sites (and all of the M7 sites) have upper 95 percent confidence limits for <1. While these K_a_/K_s _values are larger than is typical of strong purifying selection, they are markedly less than neutral expectations or even the range of K_a_/K_s _= 0.6 to 1.0 that is reported for disrupted transcribed pseudogenes in mammals [29]. Even though there appears to be selection for preserving codons, the tests cannot differentiate between past and present function. Moreover, the products need not be protein-based -- RNA interference products can elicit codon-like selection to interact with protein-coding genes [29]. The functionality and potential role of NIRVs in the well-known resistance to filoviruses of some NIRV-containing mammals (mice and guinea pigs) will have to be addressed with experiments.

**Figure 6:**
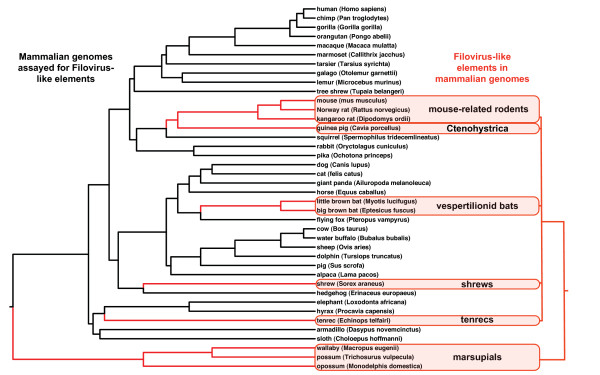
**Summary graph showing mammalian genomes assayed for filovirus-like elements and the phylogenetic distribution of the mammals with filovirus-like elements**. Red shading indicates that species with detected filovirus-like elements fall into a marsupial and a eutherian group. The platypus genome was also assayed but is not depicted here. The mammalian phylogeny is based on a composite of recent studies [30,44,45].

**Figure 7:**
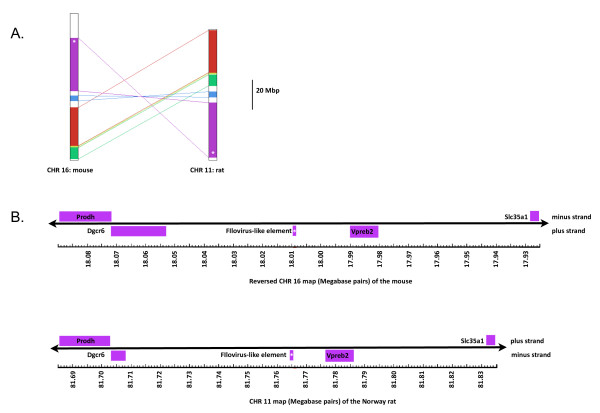
**Chromosome maps showing synteny of regions flanking filovirus-like elements in rat and mouse genomes with a whole chromosome view (A) and a local view (B)**. White asterisks represent the locations of the phylogenetic sister copies of filovirus-like elements. Five synteny blocks with a reversal distance of 2 were found between CHR 16 of the mouse and CHR 11 of the rat. The filovirus-like elements are located on a reversed synteny block (purple shading). A close up view shows the flanking gene locations and acronyms.

More than one endogenization is required to account for the paraphyly of mammals and the paraphyly of marsupials with filoviruses. The finding of a monophyletic clade for placental mammals with samples from several continents requires a single ancient integration with several losses of NIRV signal or multiple integrations of a related virus in unrelated mammal groups (Fig. 6). A single origin for eutherian NIRVs is supported by the rarity of the process -- endogenization of non-retroviral RNA viruses with extranuclear replication is previously unknown in mammals. Ancient transcribed pseudogenes >100 million years old are known from mammals [29] and the primate bornavirus integration is believed to be older than 40 million years [17]. Although much of the deeper groupings have weak support and there has been gene duplication, there are some well-supported groupings that agree with mammalian phylogeny. The strongly supported groups are the two bat genera, the genera of mouse-like rodents, and the Australian marsupials, *Trichosurus *and *Macropus*. These genera of marsupials are believed to have shared a common ancestor from 39 to 52 million years ago [30]. A clear indicator of antiquity is the syntenous genomic location of a rat and mouse filovirus-like NIRV (Fig. 7A, B). These are the same copies that have a sister group relationship (Fig. 2). It is unlikely that integration of filovirus NP genes at the same genomic position occurred independently in rats and mice. The rat-mouse orthology provides a minimum date of NIRV formation at 12 to 24 MY [31,32]. Of the species with filovirus-like elements only the rat, mouse and *Monodelphis *have detailed chromosomal maps, but further mapping and taxonomic sampling will permit a more robust assessment of the age of eutherian NIRVs. Still, we conclude that the association between filoviruses and mammals is likely to be 10's of millions of years older than the previous estimate. Filoviruses join bornaviruses as the only demonstrated prehistoric non-retroviral RNA viruses.

The eutherian orders with NIRVs of filoviruses closely match the proposed candidate reservoir groups of bats, rodents, and insectivores [1,2] (Fig. 6). This pattern is not a sampling artifact that we can attribute to the available genome assemblies. Seven of the ten genomes (including the Big Brown bat) sampled from predicted reservoir orders had integrated filoviruses, while only 1 of 27 from non-candidate eutherian orders had detected integrated filovirus-like elements (Fisher's exact test, two-tailed p value = 0.00003). The sole eutherian species from a non-candidate group to have a potential NIRV was the pygmy hedgehog tenrec, which is the Afrotherian small insectivore analog on the island of Madagascar. The three assemblies of genomes from candidate orders that lacked apparent NIRVs were the ground squirrel (*Spermophilus tridecemlineatus*), the European hedgehog (*Erinaceus europaeus*) and the fruit bat (*Pteropus vampyrus*). At present it is unclear why some small mammal groups (bats, rodents, insectivores and marsupials) appear to have an association with filoviruses. Still, the study of filovirus-like NIRVs could have predictive value for identifying filovirus reservoirs, ancestral proteins, outbreak modeling, undetected lineages of filoviruses and virulence in mammalian species. For example, the close relationship of South American and expressed Australian marsupial filovirus-like NIRVs with rapidly evolving African filoviruses now makes it more likely that the New World harbors undetected filoviruses or has acted as a source region for extant filoviruses.

## Conclusions

Our findings indicate that filovirus infections are recorded as paleoviral elements in the genomes of small mammals. These elements are candidates for functional gene products (RNA or protein). The integration is unexpected because filoviruses lack reverse transcriptase and the ability to replicate within the nucleus. Our results indicate that the association of mammals with filoviruses is likely tens of millions of years older than previously thought.

## Methods

### Nucleic Acid Extractions

DNA was extracted from freshly collected wallaby fur, toe clips of a Big Brown Bat, and DMSO preserved tissue from a little brown bat using the DNA Quickextract kit (Epicentre Technologies) modified to have a two hour incubation step at 65°C.

### PCR, RTPCR, and DNA Sequencing

50 μl PCR reactions contained 5 μL of extracted DNA template, 25 μL of 2× GoTaq PCR reagent mix (Promega) each primer. Primers for sequencing and PCR were: 5'-GCCTTGTCGACGTTCATCCTGTG-3' and 5'-GAGCCATTGGTTGCTCGGAAGC3- for *Myotis*; 5'-GGAGACCTCGAGCAAATGGAGC-3' and 5'-GAGCCATTGGTTGCTCGGAAGC-3' for *Eptesicus *and 5'-TGAGTTTTGGGGTGAATTAGC-3' and 5'-GGGTGACATAGGGAAGCACA-3' for *Macropus*. The PCR temperature profiles were: 30 cycles of 94°C for 30 s, 50°C for 30 s and 72°C for 2 min, and final extension at 72°C for 5 min. PCR products were purified and sequenced by the University of Washington High Throughput Genomics Facility. Geneious 4.8 was used to assemble and edit electrophoregrams. New sequences from this study have been named as endogenous filovirus-like NP elements (EFLNP) and assigned the following Genbank accession numbers: HM545133-HM545135.

### Bioinformatics

Initial searches for sequence similarity to filoviruses used protein sequences from genes of *Marburgvirus *(NC_001608.3) as a query with tBLASTn in the WGS database and the EST database and BLASTp in protein database of NCBI. A second tBLASTn in the same databases used the best scoring non-viral sequence of placental mammals as a query. A third search used the EST nucleotide sequences *Trichosurus *as a query for the nucleotide and WGS databases. Nonviral subject sequences with expect values of E < 10^-5 ^and two different sequences from each of the five known species in the Filoviridae were retained for alignment. A search constrained to Mononegavirales NCBI Genomic Reference Sequences Marburgvirus (NC_001608.3) found two species of *Morbillivirus *had expect values below 10^-5 ^(Rinderpest virus, and Measles virus) that were retained for alignment. L protein sequences searches used a similar strategy but many more Paramyxoviruses had a significant match to Marburgvirus. We retained 19 different Paramyxoviruses for alignment with filovirus and the mammal sequence using BLAST explorer [33].

For genome assembly sequences, the sequence boundaries and translations identified by tBLASTn were used to retrieve nucleotide sequences and assemble amino acid sequences. MAFFT [34] was used to align the protein sequences for all analyses using the default parameters. The NP alignment was trimmed to the range of the mammalian filovirus-like sequences and the L protein alignment which had a mosaic of conserved and length variable regions was trimmed by Gblocks [35] (with gaps allowed).

Phylogenetic estimates were obtained with a maximum likelihood optimality criterion (PhyML [36] and RAxML [37]) and Bayesian MCMC methods [38]. Models were chosen according to the best available optimal model from Prottest [39] (ML) or using a mixed model prior for amino acids (Mr.Bayes). Reliability was assessed by non-parametric bootstrapping (ML), approximate likelihood ratio tests (aLRT: SH like tests), and posterior probabilities. Prottest determined that the LG+G+F model was the best fit with the AIC criterion for the L protein alignment and the JTT+G model was the best fit for the NP alignment. We therefore carried out maximum likelihood analysis using these models. However, as RAxML does not accommodate the LG model we used the next best fit model of RtREV+G+F for the RAxML of L protein [40]. For bootstrapping, RAxML estimated the number of pseudoreplicates. For PhyML, both SPR and NNI search algorithms were used with five random starting trees. For Bayesian analysis, a million Markov chain Monte Carlo generations were initially carried out and convergence metrics were assessed. If the average standard deviation of split frequencies <0.01 and a plot of log-likelihood scores versus generation time as consistent with convergence, then we culled the burn-in set of half of the trees and calculated the posterior probabilities. We added 500,000 MCMC generations at a time until convergence metrics were satisfied.

Tests of neutral evolution were carried out using both approximate methods (Codon-based Z test with Kumar model [27] that accommodates transition-transversion ratio bias) and Bayesian methods [28] of estimating site-specific K_a_/K_s_. For input, codon alignments were estimated using PAL2NAL [41] from a subset of sequences from the amino acid sequence alignment. We used only one sequence per species in the alignment. As both MEGA and Selecton require continuous ORF's, disrupted codons were replaced with gaps. For the Bayesian estimate of K_a_/K_s_, an ML tree was input after estimating with PhyML and a GTR+G model. Site-specific K_a_/K_s _values were culled from the *Macropus *sequence sites, which reduced the influence of alignment end gaps on the estimates. A histogram of the K_a_/K_s _values was created in PASW statistics 18.

To evaluate orthology between rat and mouse NIRVs, we used genomic BLAST searches and visualized the matches and annotations on the NCBI chromosome maps. Whole chromosome comparisons of larger orthologous blocks were assessed using the Cinteny server [42] and Roundup database [43].

## Authors' contributions

DJT and JB conceived the study, carried out the bioinformatics analysis, participated in lab experiments and co-wrote the paper. RWL designed software and carried out BLAST searches. All authors read and approved the manuscript.

## Supplementary Material

Additional file 1 **Fig. S1**. Maximum likelihood phylogram of nucleoprotein (NP) amino acid sequences from filoviruses and marsupial sequences. Click here for file

Additional file 2 **Fig. S2**. Alignment of nucleoprotein (NP) amino acid sequences from filoviruses and related mammalian genomic and EST sequences. Disruptions to the open reading frame are shown by an "X". Click here for file

Additional file 3 **Fig. S3**. Alignment of L protein amino acid sequences (culled in Gblocks) from filoviruses and related mammalian genomic sequence. Click here for file
