# Supplementary figures and images for: Filoviruses are ancient and integrated into mammalian genomes
Source: BMC Evol Biol. 2010 Jun 22;10:193. doi: 10.1186/1471-2148-10-193 (PMC2906475; doi:10.1186/1471-2148-10-193)

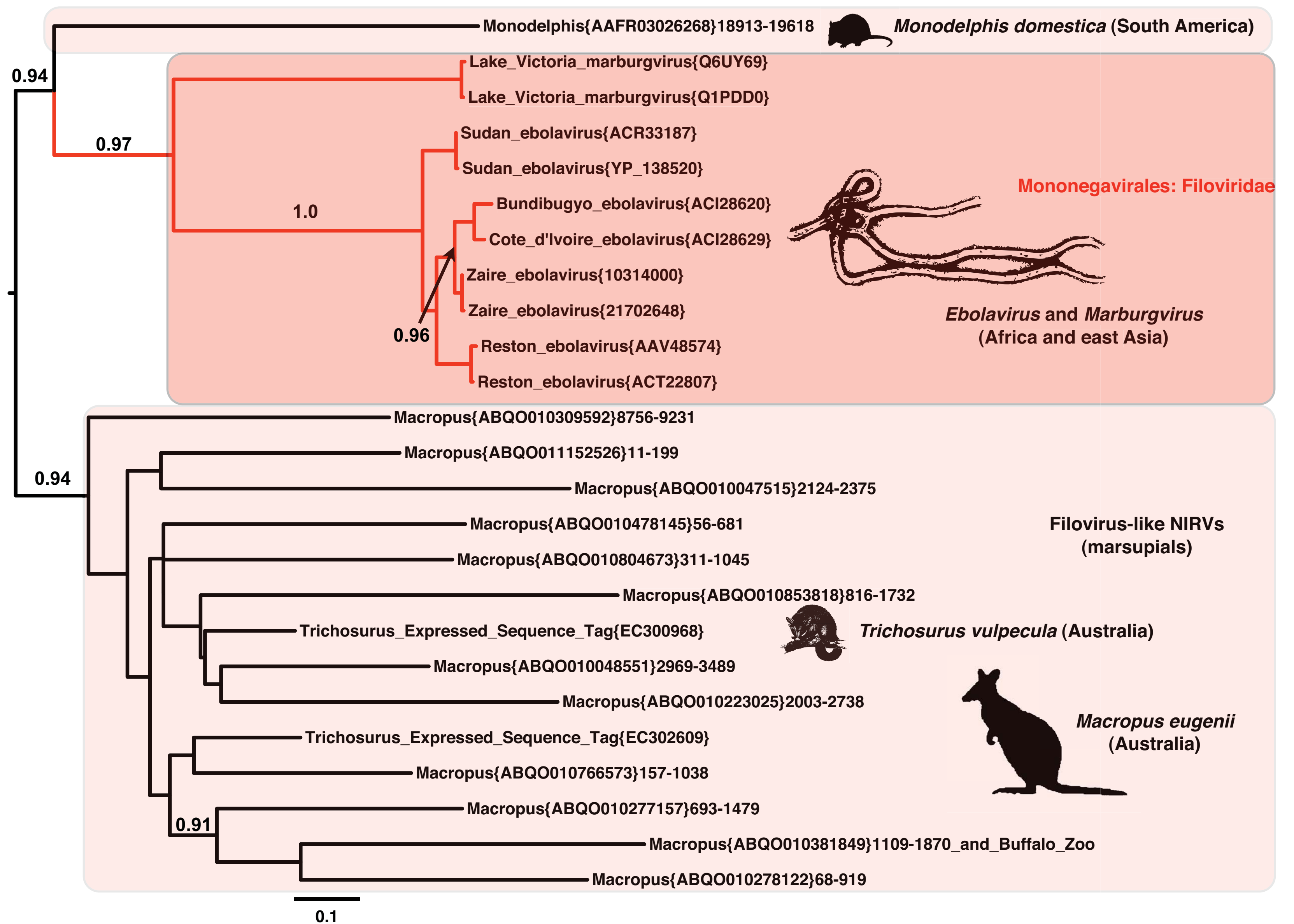

Supplement: Additional file 1 — Fig. S1. Maximum likelihood phylogram of nucleoprotein (NP) amino acid sequences from filoviruses and marsupial sequences. [file 1471-2148-10-193-S1.PDF]
