# Supplementary material for: Filoviruses are ancient and integrated into mammalian genomes
Source: BMC Evol Biol. 2010 Jun 22;10:193. doi: 10.1186/1471-2148-10-193 (PMC2906475; doi:10.1186/1471-2148-10-193)
Supplement: Additional file 2 — Fig. S2. Alignment of nucleoprotein (NP) amino acid sequences from filoviruses and related mammalian genomic and EST sequences. Disruptions to the open reading frame are shown by an "X". [file 1471-2148-10-193-S2.PDF]

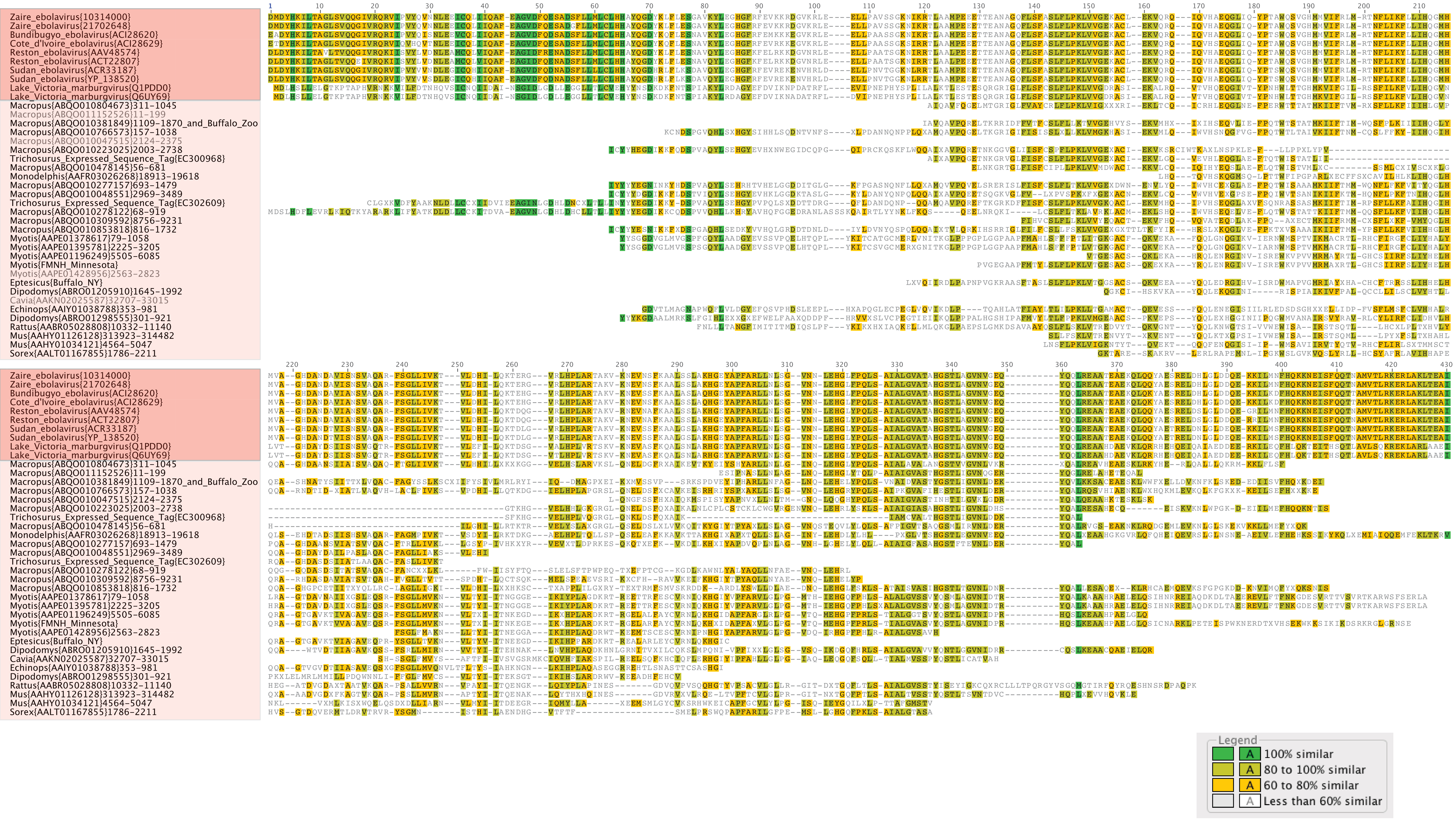
Zaire_ebolavirus{10314000}
Zaire_ebolavirus{21702648}
Bundibugyo_ebolavirus{ACI28620}
Cote_d'Ivoire_ebolavirus{ACI28629}
Reston_ebolavirus{AAV48574}
Reston_ebolavirus{ACT22807}
Sudan_ebolavirus{ACR33187}
Sudan_ebolavirus{YP_138520}
Lake_Victoria_marburgvirus{Q1PDD0}
Lake_Victoria_marburgvirus{Q6UY69}
Macropus{ABQO010804673}311-1045
Macropus{ABQO011152526}11-199
Macropus{ABQO010381849}1109-1870_and_Buffalo_Zoo
Macropus{ABQO010766573}157-1038
Macropus{ABQO010047515}2124-2375
Macropus{ABQO010223025}2003-2738
Trichosurus_Expressed_Sequence_Tag{EC300968}
Macropus{ABQO010478145}56-681
Monodelphis{AAFR03026268}18913-19618
Macropus{ABQO010277157}693-1479
Macropus{ABQO010048551}2969-3489
Trichosurus_Expressed_Sequence_Tag{EC302609}
Macropus{ABQO010278122}68-919
Macropus{ABQO010309592}8756-9231
Macropus{ABQO010853818}816-1732
Myotis{AAPE01378617}79-1058
Myotis{AAPE01395781}2225-3205
Myotis{AAPE01196249}5505-6085
Myotis{FMNH_Minnesota}
Myotis{AAPE01428956}2563-2823
Eptesicus{Buffalo_NY}
Dipodomys{ABRO01205910}1645-1992
Cavia{AAKN02025587}32707-33015
Echinops{AAIY01038788}353-981
Dipodomys{ABRO01298555}301-921
Rattus{AABR05028808}10332-11140
Mus{AAHY01126128}313923-314482
Mus{AAHY01034121}4564-5047
Sorex{AALT01167855}1786-2211
Legend
100% similar
80 to 100% similar
60 to 80% similar
Less than 60% similar
