# Supplementary material for: Filoviruses are ancient and integrated into mammalian genomes
Source: BMC Evol Biol. 2010 Jun 22;10:193. doi: 10.1186/1471-2148-10-193 (PMC2906475; doi:10.1186/1471-2148-10-193)
Supplement: Additional file 3 — Fig. S3. Alignment of L protein amino acid sequences (culled in Gblocks) from filoviruses and related mammalian genomic sequence. [file 1471-2148-10-193-S3.PDF]

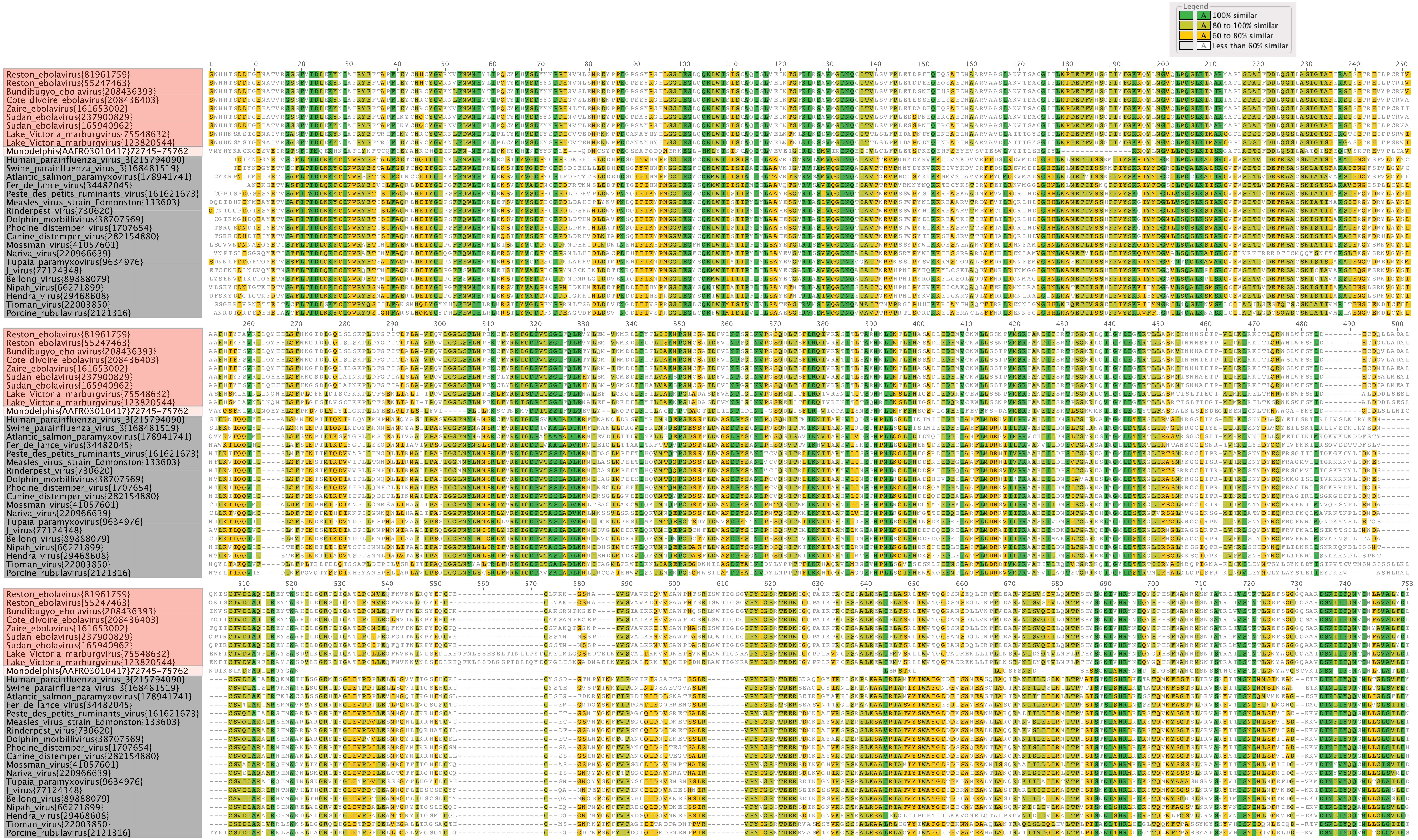
Legend
100% similar
80 to 100% similar
60 to 80% similar
Less than 60% similar
Reston_ebolavirus{81961759}
Reston_ebolavirus{55247463}
Bundibugyo_ebolavirus{208436393}
Cote_dIvoire_ebolavirus{208436403}
Zaire_ebolavirus{161653002}
Sudan_ebolavirus{237900829}
Sudan_ebolavirus{165940962}
Lake_Victoria_marburgvirus{75548632}
Lake_Victoria_marburgvirus{123820544}
Monodelphis{AAFR03010417}72745-75762
Human_parainfluenza_virus_3{215794090}
Swine_parainfluenza_virus_3{168481519}
Atlantic_salmon_paramyxovirus{178941741}
Fer_de_lance_virus{34482045}
Peste_des_petits_ruminants_virus{161621673}
Measles_virus_strain_Edmonston{133603}
Rinderpest_virus{730620}
Dolphin_morbillivirus{38707569}
Phocine_distemper_virus{1707654}
Canine_distemper_virus{282154880}
Mossman_virus{41057601}
Nariva_virus{220966639}
Tupaia_paramyxovirus{9634976}
J_virus{77124348}
Beilong_virus{89888079}
Nipah_virus{66271899}
Hendra_virus{29468608}
Tioman_virus{22003850}
Porcine_rubulavirus{2121316}
